# Genome-wide association analysis uncovers variants for reproductive variation across dog breeds and links to domestication

**DOI:** 10.1093/emph/eoz015

**Published:** 2019-05-17

**Authors:** Samuel P Smith, Julie B Phillips, Maddison L Johnson, Patrick Abbot, John A Capra, Antonis Rokas

**Affiliations:** 1 Department of Biological Sciences, Vanderbilt University, Nashville, TN 37203, USA; 2 Center for Computational Molecular Biology, Brown University, Providence, RI 02912, USA; 3 Department of Ecology and Evolutionary Biology, Brown University, Providence, RI 02912, USA; 4 Department of Biological Sciences, Cumberland University, Lebanon, TN 37087, USA; 5 Department of Biomedical Informatics, Vanderbilt University, Nashville, TN 37203, USA; 6 Vanderbilt Genetics Institute, Vanderbilt University Medical Center, Nashville, TN 37232, USA

**Keywords:** life history, artificial selection, tradeoff, preterm birth, prematurity, pregnancy

## Abstract

**Background and objectives:**

The diversity of eutherian reproductive strategies has led to variation in many traits, such as number of offspring, age of reproductive maturity and gestation length. While reproductive trait variation has been extensively investigated and is well established in mammals, the genetic loci contributing to this variation remain largely unknown. The domestic dog, *Canis lupus familiaris* is a powerful model for studies of the genetics of inherited disease due to its unique history of domestication. To gain insight into the genetic basis of reproductive traits across domestic dog breeds, we collected phenotypic data for four traits, cesarean section rate, litter size, stillbirth rate and gestation length, from primary literature and breeders' handbooks.

**Methodology:**

By matching our phenotypic data to genomic data from the Cornell Veterinary Biobank, we performed genome-wide association analyses for these four reproductive traits, using body mass and kinship among breeds as covariates.

**Results:**

We identified 12 genome-wide significant associations between these traits and genetic loci, including variants near *CACNA2D3* with gestation length, *MSRB3* and *MSANTD1* with litter size, *SMOC2* with cesarean section rate and *UFM1* with stillbirth rate. A few of these loci, such as *CACNA2D3* and *MSRB3*, have been previously implicated in human reproductive pathologies, whereas others have been associated with domestication-related traits, including brachycephaly (*SMOC2*) and coat curl (*KRT71*).

**Conclusions and implications:**

We hypothesize that the artificial selection that gave rise to dog breeds also influenced the observed variation in their reproductive traits. Overall, our work establishes the domestic dog as a system for studying the genetics of reproductive biology and disease.

**LAY SUMMARY:**

The genetic contributors to variation in mammalian reproductive traits remain largely unknown. We took advantage of the domestic dog, a powerful model system, to test for associations between genome-wide variants and four reproductive traits (cesarean section rate, litter size, stillbirth rate and gestation length) that vary extensively across breeds. We identified associations at a dozen loci, including ones previously associated with domestication-related traits, suggesting that selection on dog breeds also influenced their reproductive traits.

## INTRODUCTION

Mammals exhibit wide variation in traits associated with reproduction [1–3]. For example, gestation length can range from 12 days in the Gray dwarf hamster, *Cricetulus migratorius*, to 21 months in the African bush elephant, *Loxodonta africana* [4–6]; neonate size can range from <1 g in the shrew family (Soricidae), to more than a metric ton in the baleen whales (Balaenopteridae) [4, 6]; and neonates can be either precocial (e.g. cricetid rodents, rabbits and canids) or altricial (e.g. hystricomorph rodents, ungulates and cetaceans) [1]. This variation in reproductive traits also extends to methods of implantation [7], structure of the placenta [8, 9] and lactation strategies [10, 11]. Not surprisingly, many reproductive traits also exhibit substantial intraspecific variation [5]. For example, many mammals exhibit intraspecific variation in gestation length, including primates [12], rat and rabbits [13], as well as the domesticated cattle [14] and thoroughbred horses [15]. Similarly, body fat percentages, which are associated with the energetics of reproduction, vary greatly between wild and captive baboons, and intraspecific variation among captive lemurs can vary from 8 to 41% [16].

The existence of phenotypic variation in reproductive traits is well established, and can inform our understanding of the factors that shape patterns of survival and reproduction in both agricultural [17–20] and human populations [21]. Not surprisingly, most genome-wide association (GWAS) studies of reproductive traits focus on economically important traits in domesticated species, such as reproductive seasonality in rabbits [17], infertility in pigs [18] and dairy traits in cattle [19]. GWAS studies focused on understanding human reproductive biology and its associated pathologies have also shed light on the genetic basis of reproductive traits, including birth weight [22] and gestational duration or length [23, 24]. For example, maternal variation in six genomic loci (*ADCY5*, *AGTR2*, *EBF1*, *EEFSEC*, *RAP2C* and *WNT4*) is associated with gestational duration and preterm birth [24]. While these studies contribute to our understanding of the genetic architecture of reproductive traits, we still understand very little about the molecular pathways underlying this variation and are unable to explain the majority of the heritability in reproductive traits [25–27].

To address this challenge, we studied the genetics of reproductive traits using the domestic dog as a model system. The dog is well-suited to this question, because the domestication bottleneck followed by intense artificial selection and inbreeding imposed over the past 300 years has led to the generation of more than 340 recognized breeds that exhibit dramatic morphological variation [28–30]. Domestic dog breeds also show substantial variation in their reproductive traits. For example, Pomeranians and Norfolk Terriers typically have only 2 pups per litter, whereas Dalmatians and Rhodesian Ridgebacks typically sire 8–9 pups per litter [31]. Similarly, 80–90% of French Bulldogs and Boston Terriers are born via cesarean section due to cephalopelvic disproportion, whereas only 2–3% of Australian Shepherds and Shar Peis require cesareans [31]. Recent analyses have begun to study the genetic mechanisms that underlie the remarkable morphological variation between modern dog breeds in diverse traits such as snout length, ear erectness and tail curliness [32–35], as well as genetic disease [36].

To gain insight into the genetic basis of reproductive traits across domestic dog breeds, we collected phenotypic data for four reproductive traits, namely cesarean section rate, litter size, stillbirth rate and gestation length. We synthesized data from the primary literature and breeders’ handbooks to obtain coverage of between 23 (gestation length) and 97 (cesarean section rate) dog breeds, as well as body mass data from 101 dog breeds. By matching our phenotypic data to genome-wide genotypic data from the Cornell Veterinary Biobank, we performed GWAS analyses and identified 12 genetic loci that are significantly associated with these reproductive traits (using log body mass as a covariate). Some of these variants are in or near genes previously implicated in human reproduction-related pathologies, whereas others are in or near genes associated with domestication-related traits. For example, we found that variation in a gene previously identified to be involved in brachycephaly is also significantly associated with rates of cesarean sections and that variation in genes previously linked to coat phenotypes, such as curliness, is also associated with litter size. These results suggest that selection for breed-specific morphological traits during dog domestication may have also influenced variation in reproductive traits. More broadly, our results illustrate that the domestic dog is a promising and tractable system for studying the genetics of reproductive traits.

## METHODS

### Genotypic and phenotypic data

To identify single nucleotide polymorphisms (SNPs) that are significantly associated with reproductive traits, we used a previously published data set containing 160 727 SNPs from 4342 individual dogs across 162 breeds genotyped using the Illumina 173k CanineHD array that were downloaded from http://datadryad.org/resource/doi: 10.5061/dryad.266k4 [32]. Following the original authors, SNPs with a genotyping rate (i.e. the proportion of genotypes per marker with non-missing data) below 95% and heterozygosity ratios (i.e. the ratio of the number of heterozygous SNPs divided by the number of non-reference homozygous SNPs) below 0.25 or above 1.0 were removed. To reduce potential error stemming from SNP misidentification in our analyses, we included only SNPs with a minor allele frequency (MAF) >0.05, since SNPs with very low MAFs are more prone to error due to the small number of samples that have the called nucleotide. Application of these two filters removed 10 893 SNPs, which resulted in a final data set that contained 149 834 variants.

Phenotypic reproductive trait data for litter size (number of pups), cesarean rate, stillbirth rate and gestation length across 128 breeds were collected from a variety of breeder’s handbook and primary journal articles [31, 37–47] (see also Supplementary File S1). We also included body mass as a control trait. Each breed was assigned the average breed value for each phenotype; the full list of the values for all four reproductive traits and body mass across the 128 breeds is provided in Supplementary Table S1. For the body mass control, our collected trait values overlapped with the genotypic data [32] for 101 breeds corresponding to 3384 individuals (Table 1). For the reproductive traits, our collected cesarean section rate trait values overlapped with the genotypic data for 95 breeds (3194 individuals), our litter size trait values for 60 breeds (2617 individuals), our stillbirth rate values for 56 breeds (2590 individuals) and our gestation length values for 23 breeds (1908 individuals) (Table 2). Finally, we note that while we replicate some of the signals for the body mass GWAS from Hayward *et al.* [32], where they use individual phenotype values, we instead assigned breed averages (Supplementary Fig. S1). We performed this analysis in order to validate that breed averages are a useful proxy for individual phenotype values when testing the genetic architecture of complex traits.

**Table 1.** Numbers of breeds and individuals with overlapping phenotypes and genotypes included in our analysis

Trait	Number of overlapping breeds	Number of overlapping individuals
Body mass	101	3384
Cesarean section rate	97	3194
Litter size	60	2617
Stillbirth rate	57	2590
Gestation length	23	1908

**Table 2.** Shapiro–Wilk test of normality for the log transformed distribution of values for each phenotype

Trait	Shapiro–Wilk *P*-value
Cesarean section	0.026
Gestation length	0.491
Litter size	0.007
Stillbirth rate	0.145

### Genome-wide association analyses

To test SNPs for associations with the four reproductive traits of interest, we conducted a GWAS analysis for each individual trait using log body mass as a covariate, and accounting for kinship, as well as for body mass as a proof of concept. For each phenotype, we first log transformed all breed-average phenotype values. We then performed a Shapiro–Wilk test of normality (Table 2) for the distribution of log phenotypes for each phenotype and normalized using a Box–Cox transformation when necessary (cesarean section rate, litter size). All GWAS analyses were run using a linear-mixed model as implemented in the program GEMMA, version 0.94 [48]. Numerous studies have shown that the vast majority of morphological, ecological and physiological traits vary as a function of an organism’s body mass [49–51] as well as a function of kinship [32, 33]. Most notably for the purpose of this study, body mass has been previously shown to be strongly correlated with litter weight [52–54], neonate weight [52–55] and gestation length [6, 52, 53, 56, 57].

To ensure our analysis reflected the reproductive trait of interest and not SNPs associated with body mass, we used log body mass as a covariate for all reproductive trait analyses. To be able to do so, we pruned our genotypic data so that they included only dog breeds (and individuals) for which we had both body mass and reproductive trait of interest values (see Supplementary Table S1).

To account for population stratification, we calculated a kinship matrix of the included breeds using GEMMA and included it as a random effect in each association analysis. Each value of a kinship matrix describes the probability that a particular allele from two randomly chosen individuals at a given locus is identical by descent [58]. Finally, to control for inflated *P-*value significance from the testing of multiple hypotheses, we used a significance threshold of *P* = 3.3 × 10^−7^ (Bonferroni cutoff of *α* = 0.05, *N* = 149 834) for all analyses. All reported *P*-values are Wald’s *P*-values as calculated in GEMMA [48].

Finally, to validate variants significantly associated with at least one reproductive life history trait in the domestic dog, we performed a permutation analysis. For each trait, we randomly permuted the assignment of breed-specific phenotypes while holding body mass constant for each breed across 1000 permutations. In each permutation, we used the transformed phenotype value for each breed. Specifically, we used the log phenotype transformation for gestation length and stillbirth rate, and normalized the log phenotype transformations for cesarean section and litter size (Table 2). We then regressed the randomly assigned reproductive phenotypes onto log body mass and assigned the residual for each breed as the phenotype for all of the individuals of that breed. Next, using GEMMA [48], we performed an association test for each variant to obtain a single permuted *P*-value. The permutation *P*-value in Table 3 corresponds to the number of times that a single permuted *P*-value was less than the empirical *P*-value from the original analysis. Any variant with a permutation *P*-value >0.05 was acknowledged as a potential false positive association.

**Table 3.** Summary of genes that contain or are adjacent to the SNPs that are associated with variation in reproductive traits across dog breeds

Associated trait	Chromosome	Base pair	Mapped gene	Empirical *P*-value	Permutation *P*-value	Associated domesticated trait
Cesarean section	1	55983871	*SMOC2*	6.69e-09	0.002	Brachycephaly (Dogs)
Cesarean section	4	46570650	*MAT2B*	3.08e-07	0.001	Intramuscular preadipocyte differentiation (Pig)
Cesarean section	4	46592551	*MAT2B*	1.61e-07	0.001	Intramuscular preadipocyte differentiation (Pig)
Cesarean section	18	20272961	*CD36*	7.9e-11	0.017	
Gestation length	6	57457184	*HFM1*	1.16e-07	0.014	Fertility and milk production (Cattle)
Gestation length	20	35206774	*CACNA2D3*	3.13e-07	0.01	Blastocyst development (Cattle)
Gestation length	24	23382682		1.64e-08	0.08	
Gestation length	24	36399705	*SLC9A8*	6.75e-11	0.003	
Gestation length	24	36400728	*SLC9A8*	6.75e-11	0.003	
Gestation length	28	25752710	*PCGF5*	2.13e-07	0.003	
Litter size	1	93219668	*RCL1*	5.73e-08	0.001	
Litter size	3	61055458	*MSANTD1*	2.59e-07	0.001	
Litter size	3	61062626	*MSANTD1*	1.91e-08	0.001	
Litter size	3	61155415	*MSANTD1*	3.02e-10	0.001	
Litter size	3	61209777	*MSANTD1*	3.02e-10	0.001	
Litter size	3	61220065	*MSANTD1*	1.97e-09	0.001	
Litter size	10	7884978	*MSRB3*	3.065e-07	0.001	Ear erectness (Dog)
Litter size	10	7920882	*MSRB3*	1.27e-07	0.001	Ear erectness (Dog)
Litter size	10	8070103	*MSRB3*	1.61e-08	0.001	Ear erectness (Dog)
Litter size	10	8085469	*MSRB3*	3.55e-08	0.001	Ear erectness (Dog)
Litter size	10	8095399	*MSRB3*	5.41e-08	0.001	Ear erectness (Dog)
Litter size	10	8100127	*MSRB3*	5.41e-08	0.001	Ear erectness (Dog)
Litter size	10	8134640	*MSRB3*	2.12e-08	0.001	Ear erectness (Dog)
Stillbirth rate	25	42482266	*ENSCAFG00000010704*	1.03e-07	0.001	
Stillbirth rate	27	2539211	*UFM1*	2.01e-07	0.049	

To gain insight into the genetic elements putatively involved with the traits of interest, we mapped all SNPs found to be significantly and marginally associated with each trait of interest using custom perl and R scripts to the CanFam3.1.87 dog genome assembly [59, 60]. Transcript IDs were mapped to gene names using bioconductor biomaRt interface to the ENSEML biomart [61]. If the significant SNP was outside gene boundaries, we reported the nearest upstream or downstream gene. Manhattan plots and quantile–quantile plots were generated using R 3.1.2 [62] with the qqman package [63]. Calculation of the *λ* inflation parameter, a metric of any existing systematic bias in the data set, was calculated using the GenABEL R package [64] and was used to interpret Type I error rate in the multiple testing of GWAS analyses [65].

## RESULTS

To identify SNPs that are significantly associated with four reproductive traits in domestic dog breeds, we conducted across-breed GWAS analyses using a multivariate linear-mixed model implemented in the program GEMMA [48]. Number of individuals and distribution of breed varied with analysis (Supplementary Table S1). After filtering for MAF (MAF < 0.05; 10 804 SNPs were excluded), 149 834 ,834SNPs were included in the GWAS analysis for each reproductive trait. To control for inflated *P-*value significance from the testing of multiple hypotheses, we used a significance threshold of *P* = 3.3 × 10^−7^ (Bonferroni cutoff of *α* = 0.05, *N* = 149 834,834) for all analyses. To validate our GWAS approach and analytical choices, we first used our collected values for body mass, a trait whose genetic associations have been previously extensively studied in dogs [32]. However, whereas Hayward *et al.* [32] used individual body mass measurements in their analyses, we assigned the breed-average body mass as the phenotype for all individuals in a breed to test whether we could replicate their inferred associations for a complex trait when we used average breed value as a proxy. As expected, our analysis recovered the major genes associated with dog breed body mass variation, including *IGF1* (*P* = 2.1 × 10^−31^), *SMAD2* (*P* = 1.2 × 10^−17^) and *IGF2BP2* (*P* = 5.1 × 10^−11^) $\times 10^{-11})$(Supplementary Fig. S1 and Table S2).

### Genetic loci that significantly associate with cesarean section rate

To examine whether there is variation in cesarean section rate among breeds, we first identified cesarean section rate values for a total of 97 of the 162 dog breeds with genotypic data (Supplementary Table S1). The cesarean section rate values were derived from a British survey across 151 breeds covering 13 141 bitches, which had whelped 22 005 litters over the course of a 10 year period [30]. The frequency of cesarean sections was estimated as the percentage of litters reported to be born by cesarean section. Among the 97 breeds with overlapping genetic data, the median cesarean section rate is 17.1%, with a minimum of 0% in Curly Coated Retrievers and Silky Terriers and a maximum of 92.3% in Boston Terriers (Supplementary Fig. S3A).

**Figure 1.**
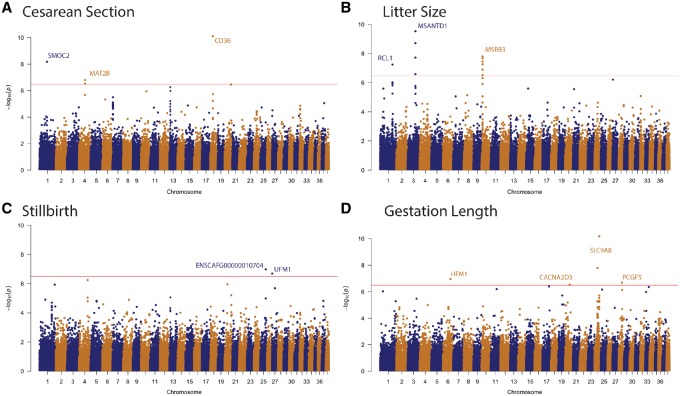
Genome-wide association results for reproductive traits in domestic dogs. Manhattan plots showing the statistical significance of each SNP as a function of genomic position for (**A**) cesarean section rate (*n* = 3, 194 individuals, *n* = 97 breeds), (**B**) litter size (*n* = 2617 individuals, *n* = 60 breeds), (**C**) stillbirth (*n* = 2590 individuals, *n* = 57 breeds) and (**D**) gestation length (*n* = 1908 individuals, *n* = 23 breeds). Horizontal line indicates the significance threshold at *P* = 4.3 × 10^−7^. Significant SNPs are labeled with the intersecting or nearest gene. Significant SNPs whose permutation *P*-values were above the 0.05 threshold. Plots were generated in R using the qqman package

To identify SNPs that are significantly associated with the observed variation in cesarean section rate across domestic dog breeds, we conducted an across-breed GWAS analysis using 149 834149,834 SNPs and cesarean section values across 95 dog breeds (Fig. 1A, Supplementary Fig. S2A). As outlined in the ‘permutation analysis’ section of the Methods, we additionally performed a breed-specific permutation and present those variants with a permutation *P*-value >0.05 as suggestive associations. We identified four significant SNPs (Supplementary Table S3), all of which mapped to genes, namely sparc-related modular calcium-binding protein 2 (*SMOC2*, uncorrected *P* = 2.0 × 10^−7^, Perm *P* = 0.011), two linked SNPs (uncorrected *P* = 3.08 × 10^−7^, Perm *P* = 0.001 and uncorrected *P* = 1.61 × 10^−7^, Perm *P* = 0.001, respectively) that mapped to *MAT2B,* and a fourth that mapped to the intergenic region between the *CD36* glycoprotein and a lincRNA (uncorrected *P* = 9.7 × 10^−8^, Perm *P* = 0.024) (Fig. 1A, Table 3).

**Figure 2.**
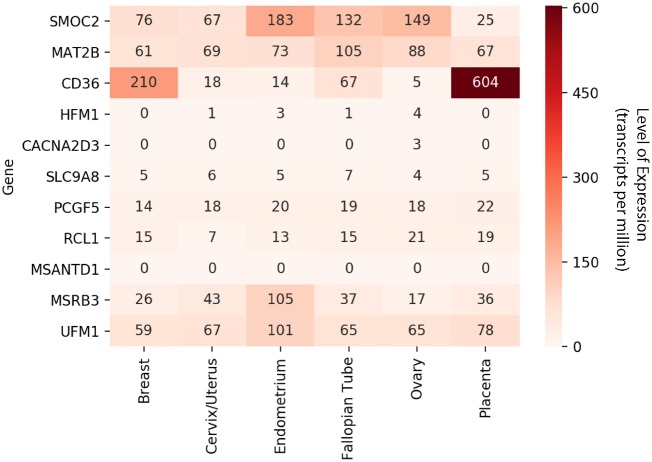
Gene expression in human female reproductive tissues of genes that contain or are adjacent to SNPs associated with reproductive traits in domestic dogs. Raw data were obtained from the Human Protein Atlas database [67]

The first significantly associated SNP is in the intron between exons 13 and 14 of *SMOC2*, a gene that is associated with dog brachycephaly and whose variation accounts for 36% of facial length variation in dogs [35, 66]. In humans, *SMOC2* is highly expressed in endometrium as well as other reproductive tissues, including the fallopian tubes, ovaries and cervix (Fig. 2) [67]. The second and third SNPs are nearby the *MAT2B* gene. Although not previously associated with reproductive traits, this gene has been shown to play a major role in intramuscular preadipocyte differentiation in domestic pigs [68]. Finally, the fourth significant SNP is in the intergenic region between the *CD36* gene and a lincRNA (ENSCAFG00000034312). The protein product of CD36 is the fourth major glycoprotein of the platelet surface and serves as a receptor for thrombospondin in platelets [69]. Other known functions include transport of long chain fatty acids [70]. However, we believe that this variant is more likely a replication of a previous association between the nearby *FGF4* retrotransposon and chondrodysplasia across breeds [71].

### Genetic loci that significantly associate with litter size

To identify SNPs that are significantly associated with variation in litter size among breeds, we retrieved litter size data from 10 810 litters of 224 breeds registered in the Norwegian Kennel Club [37]. For these data, we were able to obtain average number of pups per litter values for 60 of the 162 dog breeds with overlapping genetic data (Supplementary Table S1). Among these 60 breeds, median litter size is 5.55 pups, with a maximum 8.9 in Rhodesian Ridgebacks and a minimum of 2.4 in Pomeranians (Supplementary Fig. S3B).

To identify SNPs that are significantly associated with the observed variation in litter size across domestic dog breeds, we conducted an across-breed GWAS analysis using 149 834 149,834SNPs and litter size data from 60 dog breeds (Fig. 1B, Supplementary Fig. S2B). We identified three independently associated regions containing 13 SNPs (Table 3, Supplementary Table S4) intersecting three genes, namely RNA Terminal Phosphate Cyclase-Like 1 (*RCL1*, uncorrected *P* = 2.6 × 10^−8^, Perm *P* = 0.001), Myb/SANT SNA Binding Domain Containing 1 (*MSANTD1*, see Table 3 for a full list of nearby linked SNPs and their association statistics) and methionine sulfoxide reductase B3 (*MSRB3*, see Table 3 for a full list of nearby linked SNPs and their association statistics).

The *RCL1* SNP is found in the intron between exons 7 and 8. *RCL1* functions in the maturation of 18s RNA [72] and is associated with cervical cancer; one role of the gene in this cancer pathology is thought to involve the regulation of insulin receptors [72]. Additionally, a rare missense variation in *RCL1* was recently associated with depression [73]. The second SNP in the intergenic region downstream of *MSRB3*, whose protein product catalyzes the reduction of methionine-R-sulfoxides to methionine and repairs oxidatively damaged proteins [74, 75]. In humans, *MSRB3* mutations are associated with deafness [76]. Epigenetic changes of *MSRB3* in the fetus during pregnancy may affect length of gestation, with increased DNA methylation correlated with increased gestational age [77, 78]. Furthermore, *MSRB3* shows an increase in mRNA expression in ripe (at term) versus unripe human uterine cervix, implying that *MSRB3* functions to ripen the cervix before the onset of labor [79]. In previous morphological studies in dogs, *MSRB3* is associated with ear erectness [33]. We additionally point out that this association is nearby the *HMGA2* gene that has been previously associated with body mass in several different organisms (domestic dog [32], pigs [80], mice [81, 82]). The last SNP that is significantly associated with litter size is located downstream of *MSANTD1*, which is part of a gene network believed to aid in cell-to-cell signaling and interaction, hematological system development and function and immune cell trafficking [83]. *MSANTD1* has been identified in two independent studies as a candidate gene for the determination of black coat color in goats [84, 85].

### Genetic loci that significantly associate with stillbirth rate

To examine whether there are SNPs that are significantly associated with variation in stillbirth rate among breeds, we retrieved data for stillbirth rates for 57 of the 162 dog breeds (Supplementary Table S1). The data covers 10 810 litters of 224 breeds registered in the Norwegian Kennel Club and defines perinatal mortality as the sum of stillborn puppies and puppies that died during the first week after birth [38]. Among these 57 breeds with overlapping genomic data, the median stillbirth rate is 4.2 pups, with a maximum rate of 12.3% in Saint Bernards and a minimum of 0% in Basenjis and Italian Greyhounds (Supplementary Fig. S3C).

To test if any SNPs are significantly associated with the observed variation in stillbirth rate across domestic dog breeds, we conducted an across-breed GWAS analysis using 149 834 SNPs and stillbirth rate data from 56 dog breeds (Fig. 1C, Supplementary Fig. S2C). We identified two significantly associated SNPs (Table 3, Supplementary Table S5); both intersect genes, Ubiquitin fold modifier 1 (*UFM1*, uncorrected *P* = 2.01 × 10^−7^, Perm *P* = 0.049) and near a novel gene (ENSCAFG0000010704, uncorrected *P* = 1.03 × 10^−7^, Perm *P* = 0.001). The *UFM1* SNP has been previously associated with coat color variation in the domestic dog [86], but not with reproductive life history trait variation.

### Genetic loci that significantly associate with gestation length

To examine whether there is variation in gestation length among breeds, we identified individual gestation length averages by breed predominantly in breeder handbooks. Utilizing breeders’ handbooks, we were able to identify gestation length means for a total of 23 of the 162 dog breeds that we had genotypic data for (Supplementary Table S1). Among these 23 breeds, the median gestation length is 62.2 days, with a maximum length of 65.3 in beagles and a minimum of 60.1 in the Alaskan Malamute (Supplementary Fig. S3D).

To identify SNPs significantly associated with the observed variation in gestation length across domestic dog breeds, we conducted an across-breed GWAS analysis using 149 834 SNPs and gestation length data from 23 dog breeds (Fig. 1D, Supplementary Fig. S2D). Our analysis identified five significantly associated SNPs (Table 3, Supplementary Table S6) that mapped to four genes, namely solute carrier family 9 (*SLC9A8*, uncorrected *P* = 3.7 × 10^−11^, Perm *P* = 0.001), calcium channel, voltage-dependent, alpha-2/delta Subunit 3 (*CACNA2D3*, uncorrected *P* = 3.1 × 10^−7^, Perm *P* = 0.013 and polycomb group ring finger 5 (*PCGF5, P* = 6.75 × 10^−11^, Perm *P* = 0.003).

The first SNP resides in intron 78 of *SLC9A8*, an integral transmembrane protein that exchanges extracellular Na^+^ for intracellular H^+^. *SLC9A8* serves multiple functions [87] and is expressed ubiquitously (Fig. 2) [67]. Knockout male mice have impaired luteinizing hormone-stimulated cAMP production and are infertile, despite normal morphology of their reproductive system and normal behavior [88]. The second SNP is found in the intron between exons 26 and 27 of *CACNA2D3*. This gene is involved in regulating the influx of Ca^2+^ ions entering the cell upon membrane polarization [89], a critical process relevant to many functions, including fertilization and development [90]. In previous studies in humans, *CACNA2D3* is differentially methylated in the amnion between normal and pre-eclamptic pregnancies [91] and in blood between extreme preterm and term infants at birth [92, 93]. Additionally, *CACNA2D3* is one of four genes recently described as influencing cranial morphology in human populations [94]. In other domesticated animals, *CACNA2D3* is downregulated by Colony Stimulating Factor 2 (*CSF2*) in the trophectoderm of pregnant cattle, which increases the ability of the preimplantation embryo to advance to the blastocyst stage [95]. In the closely related wolf, *CACNA2D3* is under diversifying selection associated with environmental adaptations to altitude [96–98]. Finally, the last SNP mapped to *PCGF5*, a gene previously shown to be necessary for neural differentiation in embryonic growth cell in humans [99].

## DISCUSSION

Mammals exhibit a great deal of variation in their reproductive traits, yet remarkably little is known about the genetic basis of these traits. To begin to address this, we used GWAS analyses to examine the genetic basis of four reproductive traits (cesarean section rate, stillbirth rate, litter size and gestation length) across up to 97 domestic dog breeds. We identified several significant genetic associations for each trait (Fig. 1, Table 3).

Four of the 12 genetic regions that we found to be associated with reproductive trait variation have been previously identified to be involved in diverse traits associated with dog domestication (Table 3), such as brachycephaly and coat curl and color, suggesting that selection for signature traits of dog breeds may have also directly or indirectly influenced variation in reproductive traits. For example, one of the variants that we found to be associated with cesarean section rate is in an intron of *SMOC2*, a gene previously associated with brachycephaly in dogs [35, 66]. Brachycephaly, the shortening and widening of the muzzle and skull, is present in several ‘fighting’ breeds such as Boxer, Boston Terrier and Bulldog, and is thought to have been originally artificially selected on the basis that a shorter and wider cranial shape would enhance the dog’s biting power [100]. Interestingly, one of the traits that associated with brachycephaly is cephalopelvic disproportion [31], a significant medical condition that can result in the death of both the litter and the bitch due to the inability of the pups to pass through the pelvic canal. The negative effects of cephalopelvic disproportion are alleviated by cesarean section, which not only allows these breeds to reproduce but also enables the continued application of artificial selection for the most extreme cranial morphology [66]. Whether the *SMOC2* variant identified directly influences parturition and birth timing in dogs (in humans, *SMOC2* is highly expressed in several reproductive tissues; see Fig. 2 and Ref. [67] or indirectly leads to adverse pregnancy outcomes (e.g. brachycephalic cranial morphology leading to cesarean section) remains unknown. It is highly likely, however, that the association between *SMOC2* and brachycephaly came first, paving the way for the subsequent association of both with cesarean section rate.

Several of the significantly associated genes that we identified in dogs appear to also be associated with reproductive phenotypes in humans. This suggests the possibility that the artificial selection that gave rise to dog breeds may have also contributed to the observed variation in their reproductive traits. For example, a member of the gene family for a subunit of the voltage-dependent calcium channel complex, *CACNA2D3,* which is associated with gestation length in our study, has been shown to be both differentially methylated in amnion between normal and pre-eclamptic human pregnancies [91], and in blood between extreme preterm and term infants at birth [92, 93]. Similarly, expression of *MSRB3*, which is associated with litter size in our study, is elevated in ripe (at term) versus unripe human uterine cervix and may be involved in the onset of labor [78], and *SMOC2* is also known to be expressed in human reproductive tissues (Fig. 2) [67].

There are several caveats to our analyses and results. To begin with, as is true of all GWAS studies, our findings will need to be replicated in other cohorts and our hypotheses about the variants and genes involved will need to be functional validated. Furthermore, our set of reproductive phenotype data is breed averages rather than individual values, which would allow more precise association analyses. Additionally, it should be noted that some of the values of our reproductive phenotypic data may not be always precise. For example, there is likely variability across breeds for total litter size and ultimately 1 week viability (stillbirth). Similarly, the cesarean section phenotype is largely determined by the owner and the veterinarian. In this way, it is an elective phenotype measurement but is informed by the breed-specific likelihood of a healthy delivery. However, cesarean section rates for some breeds are extremely high (e.g. 92.3% in Boston terrier), which seems to indicate that cesarean section procedures are necessary to preserve the health of both the mother and the pups. Therefore, we assume that these rates reflect veterinarians’ information and knowledge of potential breed-specific birthing risks.

Previous analyses of traits, such as body mass, in the domestic dog have found that much of the variation can be explained by a few large-effect loci [101, 102]. While our results show that our approach and use of breed-specific average trait values are sufficiently powered to detect traits determined by a few causal variants of large effects, we still lack knowledge of the underlying genetic architecture of the four reproductive traits analysed in this study. The results of a recent GWAS of gestation length in humans [24] suggest that, at least in humans, variation in gestation length is likely explained by many small-effect variants; if that is the case, approaches based on breed averages may fail to detect variants of low effect due to the lack of sufficient phenotypic stratification. Finally, as is true for most complex traits in the domestic dog, the reproductive traits studied here are likely to have also been influenced by the rapid and strong artificial selection imposed during the recent breeding history of the domestic dog.

## Supplementary Material

eoz015_Supplementary_Data
