# Supplementary material for: Genome-wide association analysis uncovers variants for reproductive variation across dog breeds and links to domestication
Source: Evol Med Public Health. 2019 May 17;2019(1):93–103. doi: 10.1093/emph/eoz015 (PMC6592264; doi:10.1093/emph/eoz015)
Supplement: eoz015_Supplementary_Data [file eoz015_supplementary_data.zip › Smith_etal_FileS1_PhenotypeResources.docx]

**Supplementary File 1. Sources of phenotypic data.**

**Body Mass**

All Body Mass data points were taken from: Evans, Jim M., and Kay White. The book of the bitch: a complete guide to understanding and caring for bitches. Interpet, 1997. In cases where a range was provided, the average of the two values was assigned as the phenotype.

**Litter Size**

All Litter Size data points for 66 breeds were taken from the mean column in **Table 1** of: Borge, Kaja Sverdrup, et al. "Litter size at birth in purebred dogs—A retrospective study of 224 breeds." Theriogenology 75.5 (2011): 911-919.

**Cesarean Section Rate**

All Cesarean Section Rate data points for 116 breeds were taken from the percentage column of **Table 1** of: Evans, Katy M., and Vicki J. Adams. "Proportion of litters of purebred dogs born by caesarean section." Journal of small animal practice 51.2 (2010): 113-118.

**Stillbirth Rate**

All Stillbirth Rate data points for 66 breeds were taken from the Puppy Level section, percentage column of **Table 2** of: Tønnessen, R., et al. "Canine perinatal mortality: a cohort study of 224 breeds" Theriogenology 77.9 (2012): 1788-1801.

**Gestation Length**

The data points for gestation length were taken from a variety of sources, these sources are listed with the corresponding breed in the table below.

| **Breed** | **Gestation Length (Days)** | **Citation** |
| --- | --- | --- |
| Alsatians | 60.1 | Okkens AC, Hekerman TWM, de Vogel JWA, van Haaften B. Influence of litter size and breed on variation in length of gestation in the dog. Vet Q. 1993 Dec;15(4):160–1. |
| Basenji | 62.18 | Linde Forsberg C, Wikström C, Lundeheim N. Matings by season. [cited 2017 Jun 28]; Available from: http://canirep.com/res/CaniRepDoc/season.pdf |
| Beagle | 65.3 | Concannon P, Whaley S, Lein D, Wissler R. Canine gestation length: variation related to time of mating and fertile life of sperm. Am J Vet Res. 1983 Oct;44(10):1819–21. |
| Bernese Mountain Dog | 61.7 | Okkens AC, Hekerman TWM, de Vogel JWA, van Haaften B. Influence of litter size and breed on variation in length of gestation in the dog. Vet Q. 1993 Dec;15(4):160–1. |
| Bouvier des flandres | 62.3 | Okkens AC, Hekerman TWM, de Vogel JWA, van Haaften B. Influence of litter size and breed on variation in length of gestation in the dog. Vet Q. 1993 Dec;15(4):160–1. |
| Boxer | 62.4 | Okkens AC, Hekerman TWM, de Vogel JWA, van Haaften B. Influence of litter size and breed on variation in length of gestation in the dog. Vet Q. 1993 Dec;15(4):160–1. |
| Cavalier King Charles Spaniel | 61.11 | Linde Forsberg C, Wikström C, Lundeheim N. Matings by season. [cited 2017 Jun 28]; Available from: http://canirep.com/res/CaniRepDoc/season.pdf |
| Cocker Spaniel | 61.95 | Linde Forsberg C, Wikström C, Lundeheim N. Matings by season. [cited 2017 Jun 28]; Available from: http://canirep.com/res/CaniRepDoc/season.pdf |
| Doberman Pinscher | 61.4 | Okkens AC, Hekerman TWM, de Vogel JWA, van Haaften B. Influence of litter size and breed on variation in length of gestation in the dog. Vet Q. 1993 Dec;15(4):160–1. |
| Swedish Drever | 61.48 | Gavrilovic BB, Andersson K, Linde Forsberg C. Reproductive patterns in the domestic dog—A retrospective study of the Drever breed. Theriogenology. 2008 Sep;70(5):783–94. |
| English Setter | 62.19 | Linde Forsberg C, Wikström C, Lundeheim N. Matings by season. [cited 2017 Jun 28]; Available from: http://canirep.com/res/CaniRepDoc/season.pdf |
| German Sheperd | 63.6 | Eilts BE, Davidson AP, Hosgood G, Paccamonti DL, Baker DG. Factors affecting gestation duration in the bitch. Theriogenology. 2005 Jul;64(2):242–51 |
| Golden Retriever | 64.7 | Eilts BE, Davidson AP, Hosgood G, Paccamonti DL, Baker DG. Factors affecting gestation duration in the bitch. Theriogenology. 2005 Jul;64(2):242–51 |
| Labrador Retriever | 62.9 | Eilts BE, Davidson AP, Hosgood G, Paccamonti DL, Baker DG. Factors affecting gestation duration in the bitch. Theriogenology. 2005 Jul;64(2):242–51 |
| Maltese | 63.2 | Son C, Jeong K, Kim J, Park I, Kim S, Lee C. Establishment of the Prediction Table of Parturition Day with Ultrasonography in Small Pet Dogs. J Vet Med Sci. 2001;63(7):715–21. |
| Norwegian Elkhound | 62.09 | Linde Forsberg C, Wikström C, Lundeheim N. Matings by season. [cited 2017 Jun 28]; Available from: http://canirep.com/res/CaniRepDoc/season.pdf |
| Norwich/Norfolk Terrier | 61.28 | Linde Forsberg C, Wikström C, Lundeheim N. Matings by season. [cited 2017 Jun 28]; Available from: http://canirep.com/res/CaniRepDoc/season.pdf |
| Ole English Sheepdog | 62 | Okkens AC, Hekerman TWM, de Vogel JWA, van Haaften B. Influence of litter size and breed on variation in length of gestation in the dog. Vet Q. 1993 Dec;15(4):160–1. |
| Pug | 61.58 | Linde Forsberg C, Wikström C, Lundeheim N. Matings by season. [cited 2017 Jun 28]; Available from: http://canirep.com/res/CaniRepDoc/season.pdf |
| Rottweiler | 60.2 | Chatdarong K, Tummaruk P, Sirivaidyapong S, Raksil S. Seasonal and breed effects on reproductive parameters in bitches in the tropics: a retrospective study. J Small Anim Pract. 2007 Aug;48(8):444–8. |
| Miniature Schnauzer | 63 | Kim B-S, Son C-H. Time of initial detection of fetal and extra-fetal structures by ultrasonographic examination in Miniature Schnauzer bitches. J Vet Sci. 8(3):289–93 |
| Soft-coated Wheaten | 62.06 | Linde Forsberg C, Wikström C, Lundeheim N. Matings by season. [cited 2017 Jun 28]; Available from: http://canirep.com/res/CaniRepDoc/season.pdf |
| Weimaraner | 62.8 | Okkens AC, Hekerman TWM, de Vogel JWA, van Haaften B. Influence of litter size and breed on variation in length of gestation in the dog. Vet Q. 1993 Dec;15(4):160–1. |
| Yorkshire Terrier | 63.4 | Son C, Jeong K, Kim J, Park I, Kim S, Lee C. Establishment of the Prediction Table of Parturition Day with Ultrasonography in Small Pet Dogs. J Vet Med Sci. 2001;63(7):715–21. |
