# Supplementary figures and images for: Genome-wide association analysis uncovers variants for reproductive variation across dog breeds and links to domestication
Source: Evol Med Public Health. 2019 May 17;2019(1):93–103. doi: 10.1093/emph/eoz015 (PMC6592264; doi:10.1093/emph/eoz015)

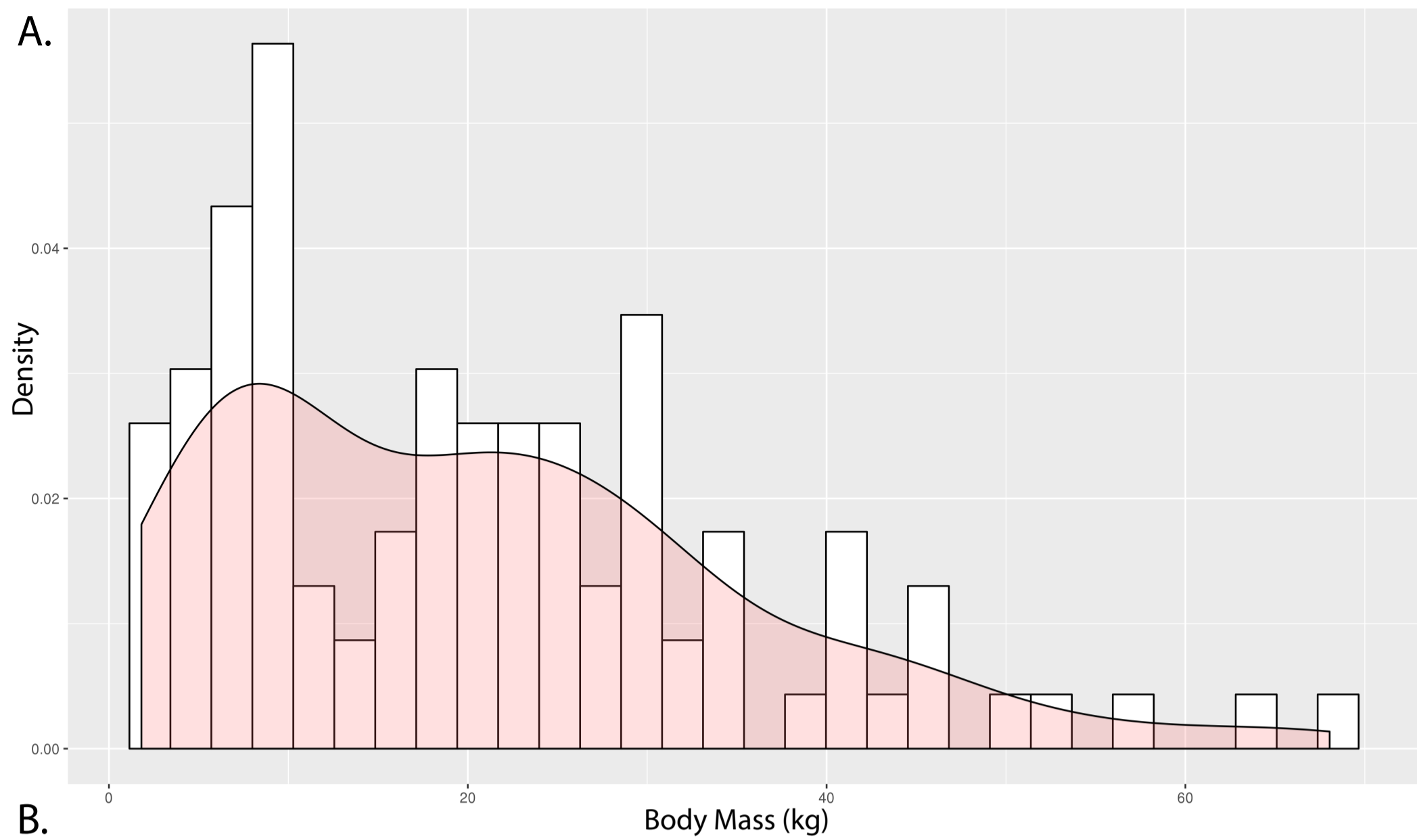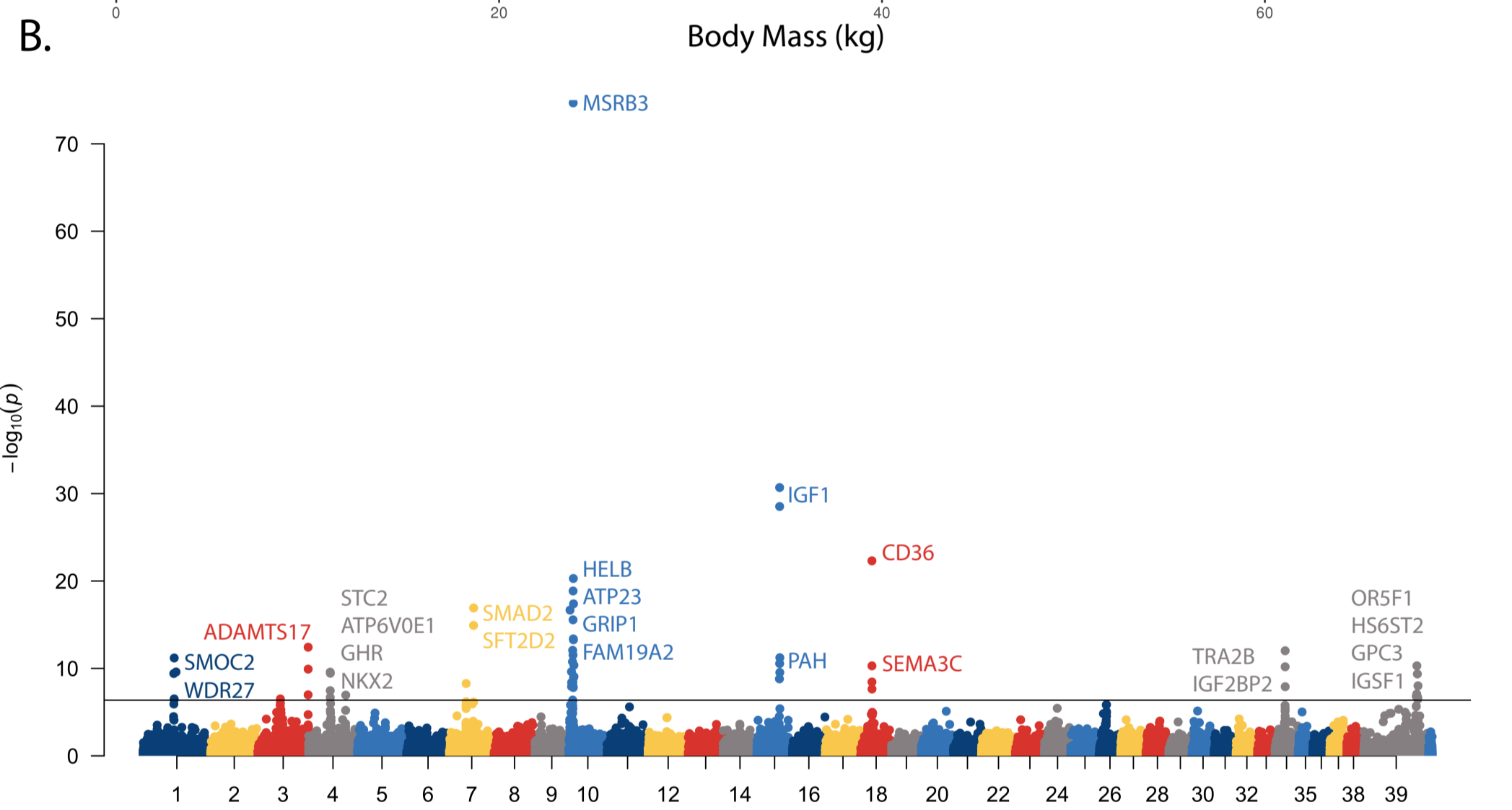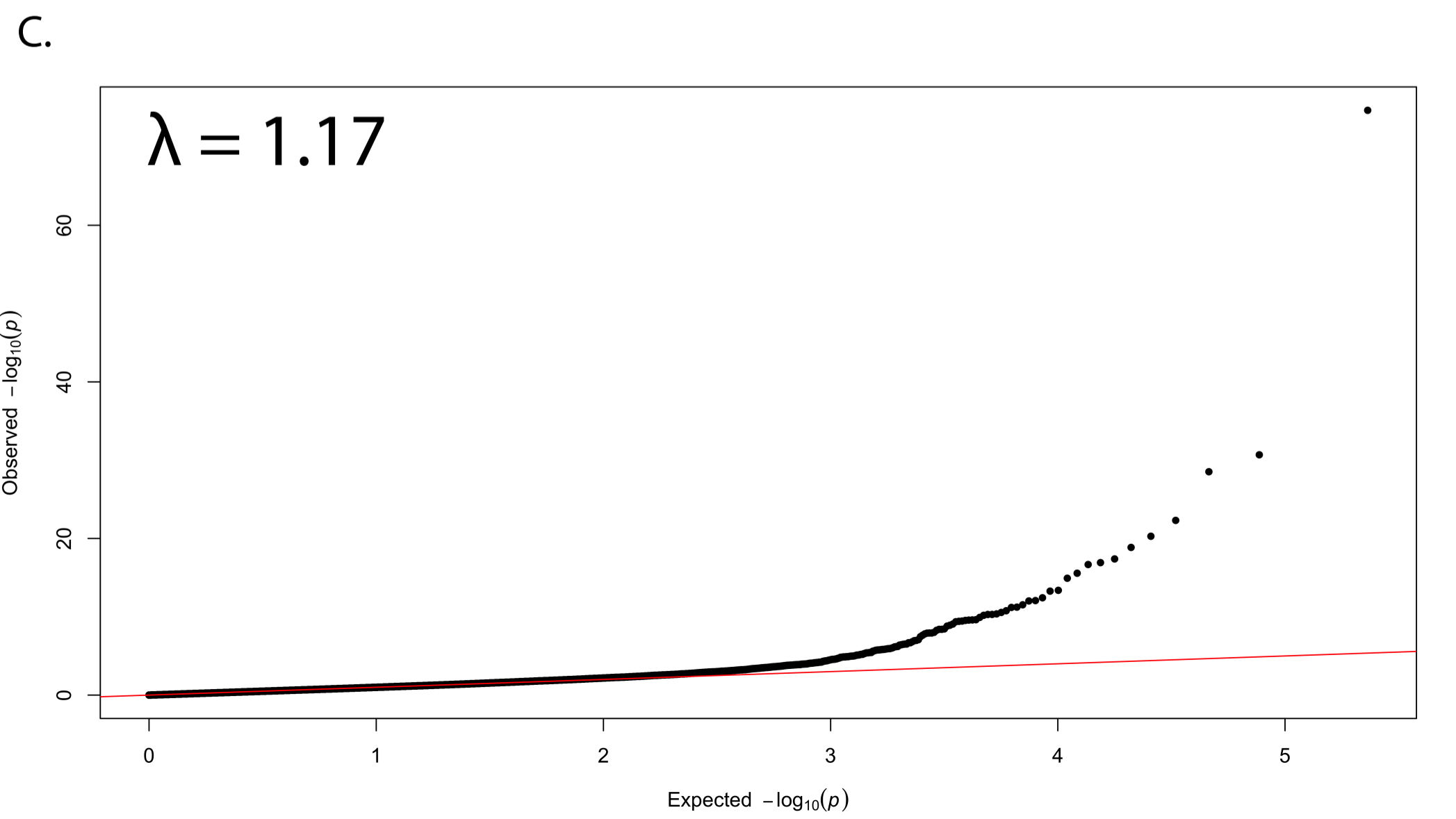

Supplement: eoz015_Supplementary_Data [file eoz015_supplementary_data.zip › Smith_etal_FigS1_BodyMass.pdf]

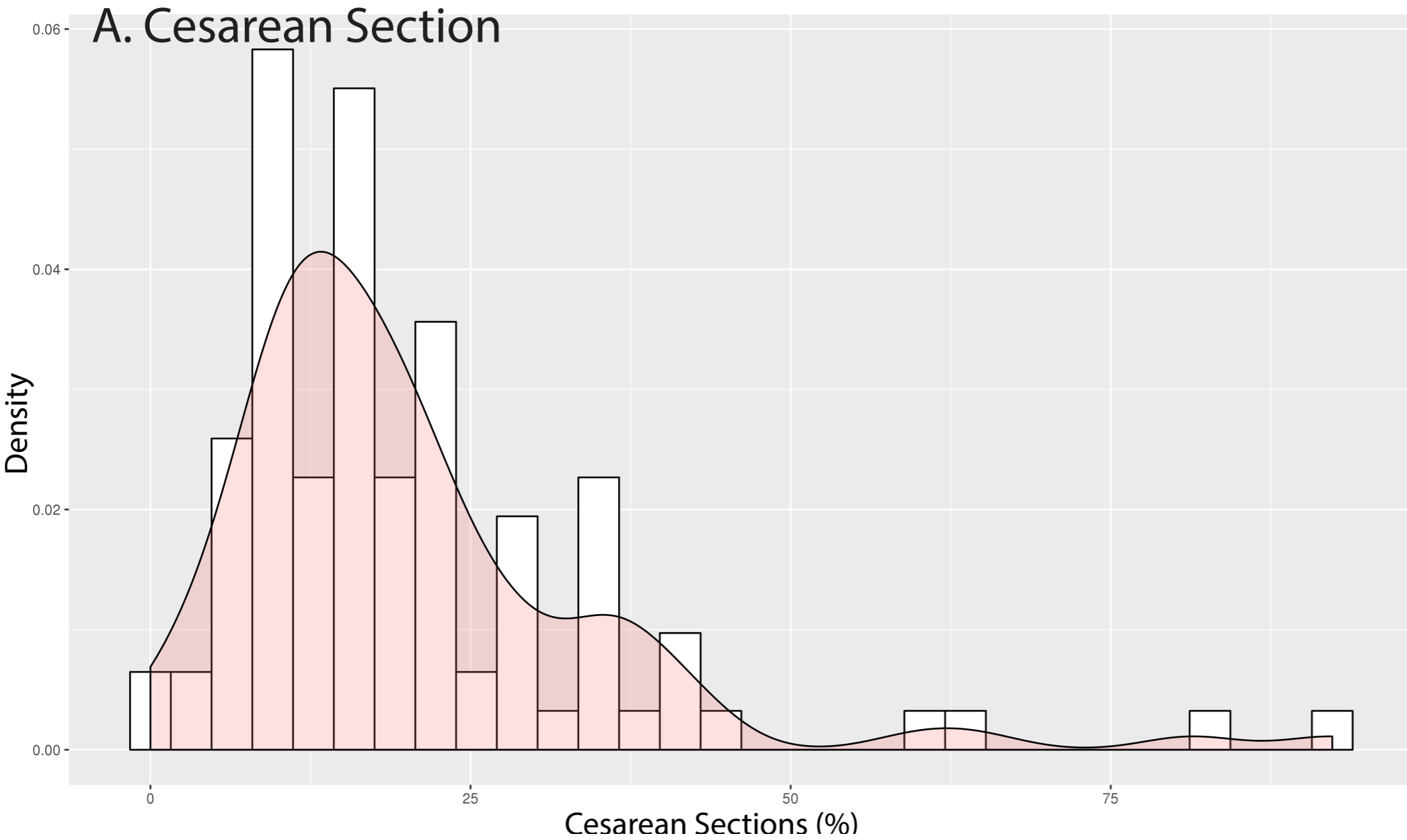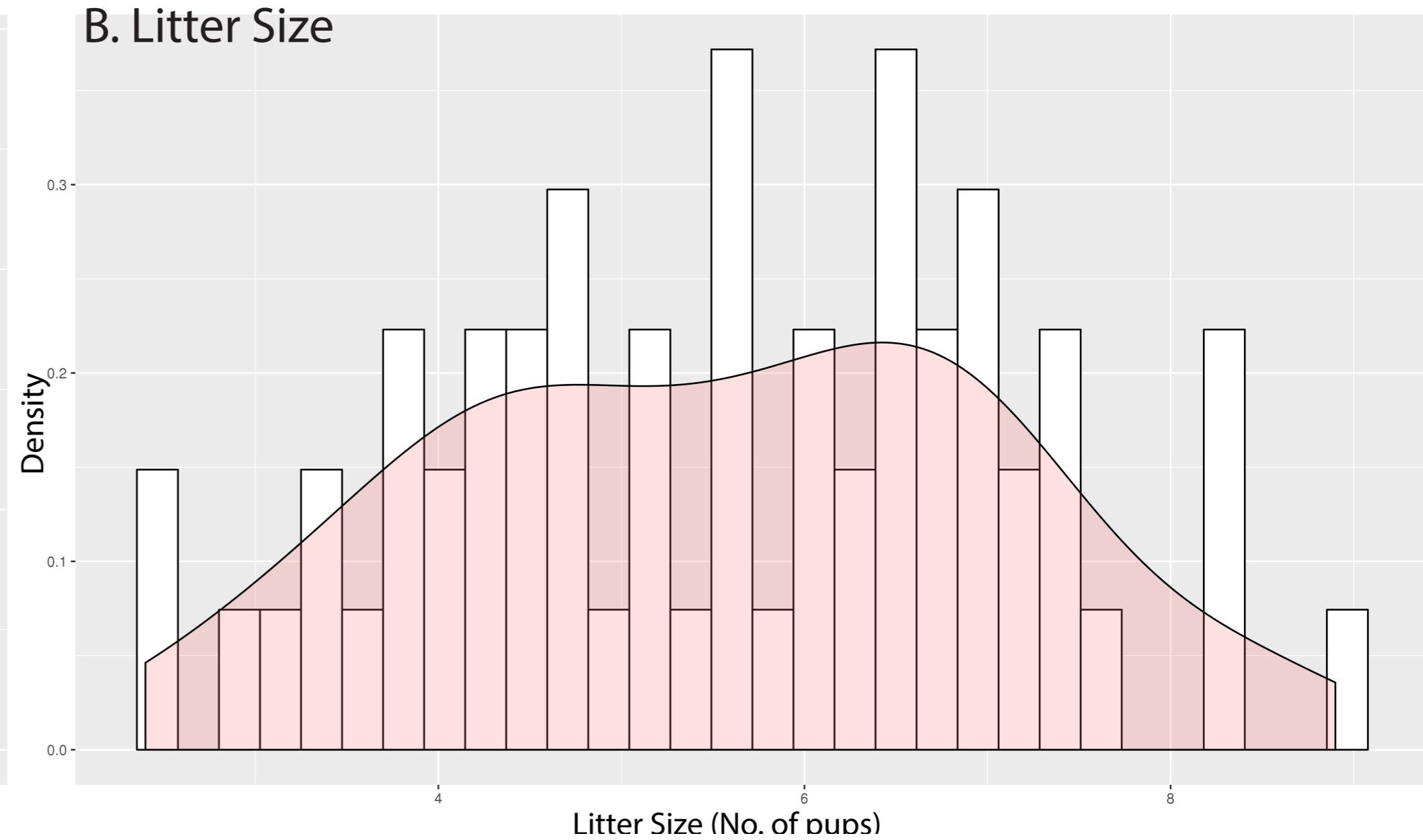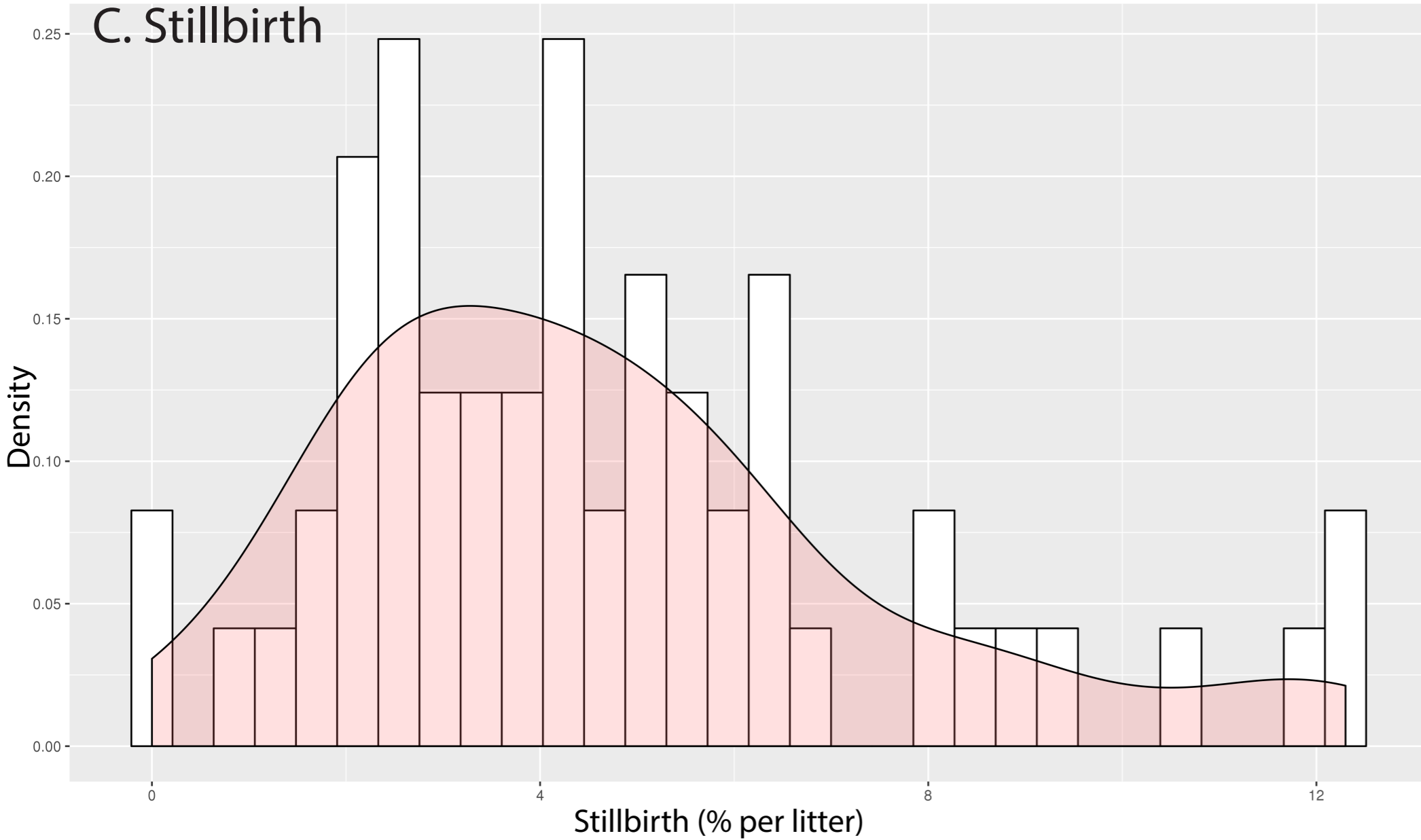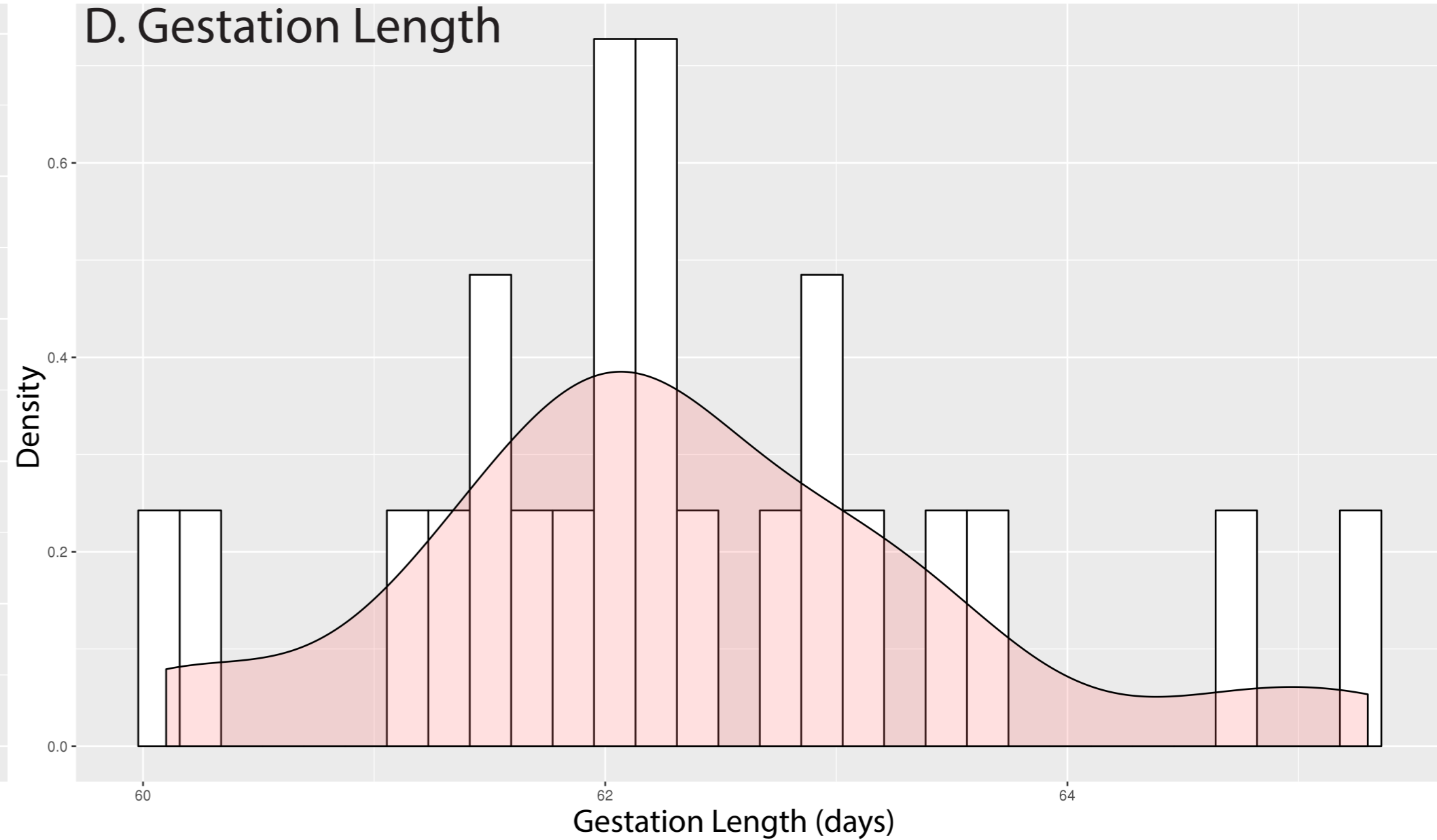

Supplement: eoz015_Supplementary_Data [file eoz015_supplementary_data.zip › Smith_etal_FigS2_TraitsDistribution.pdf]

# A. Cesarean Section

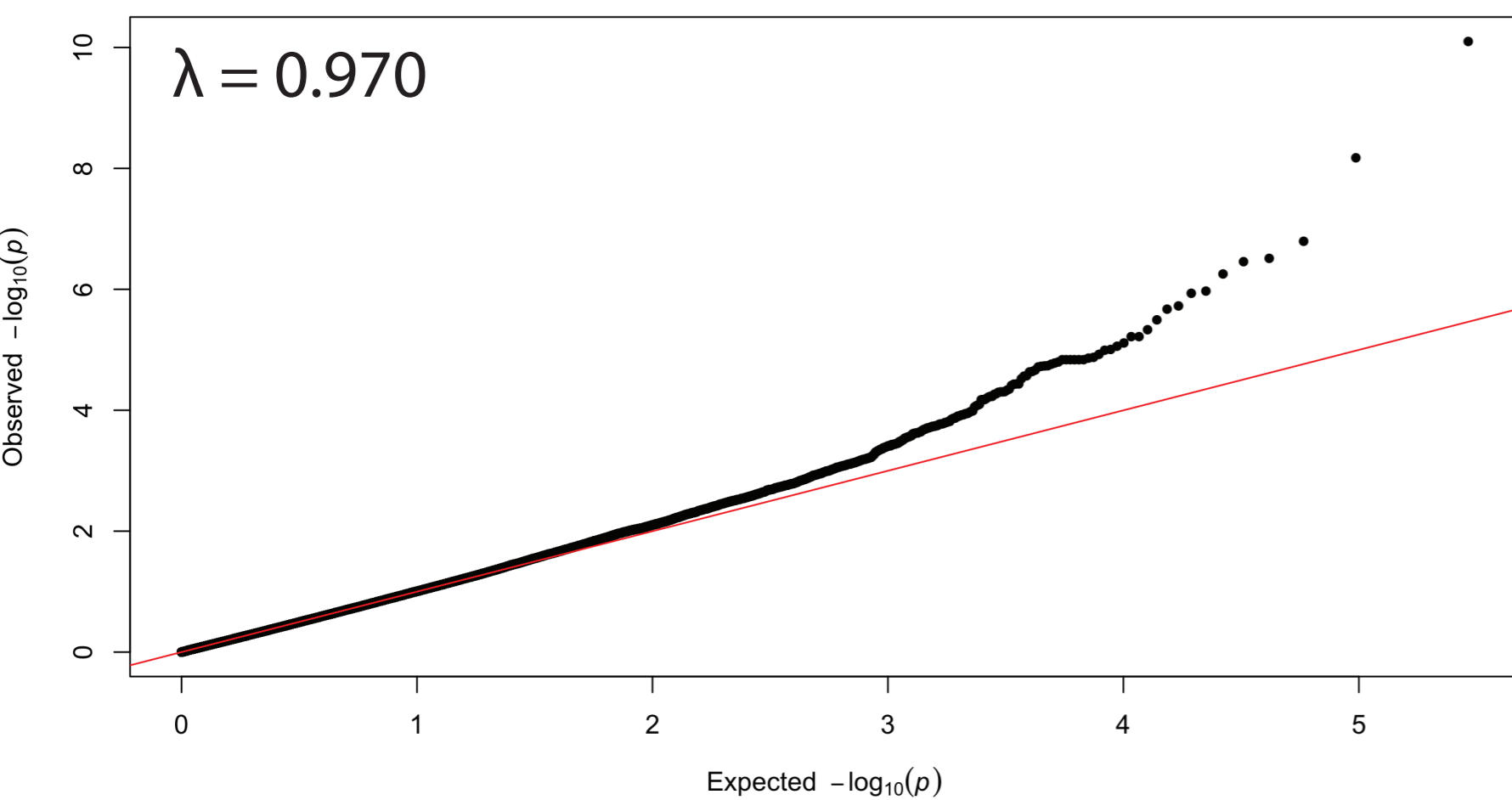

# B. Litter Size

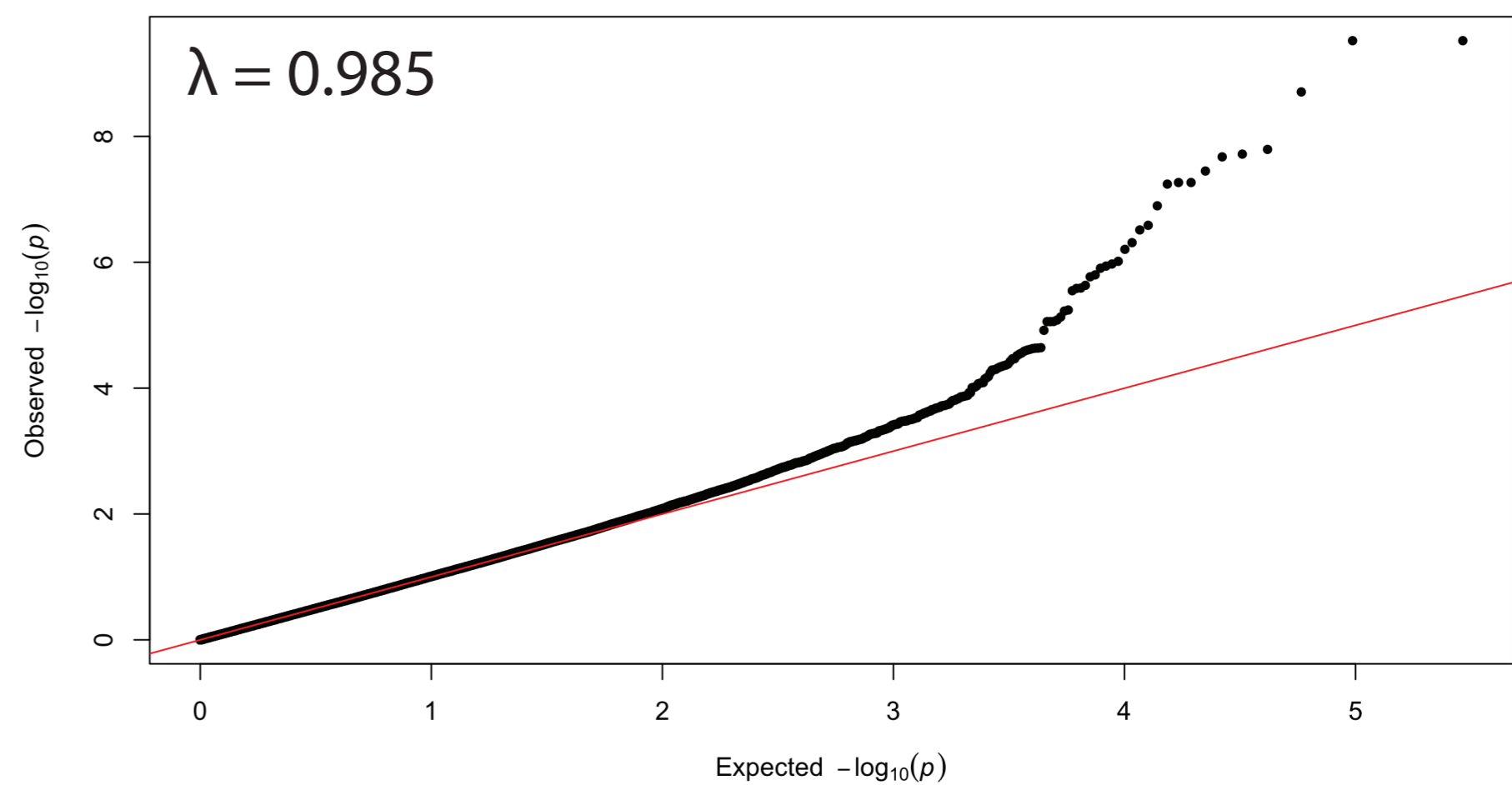

# C. Stillbirth

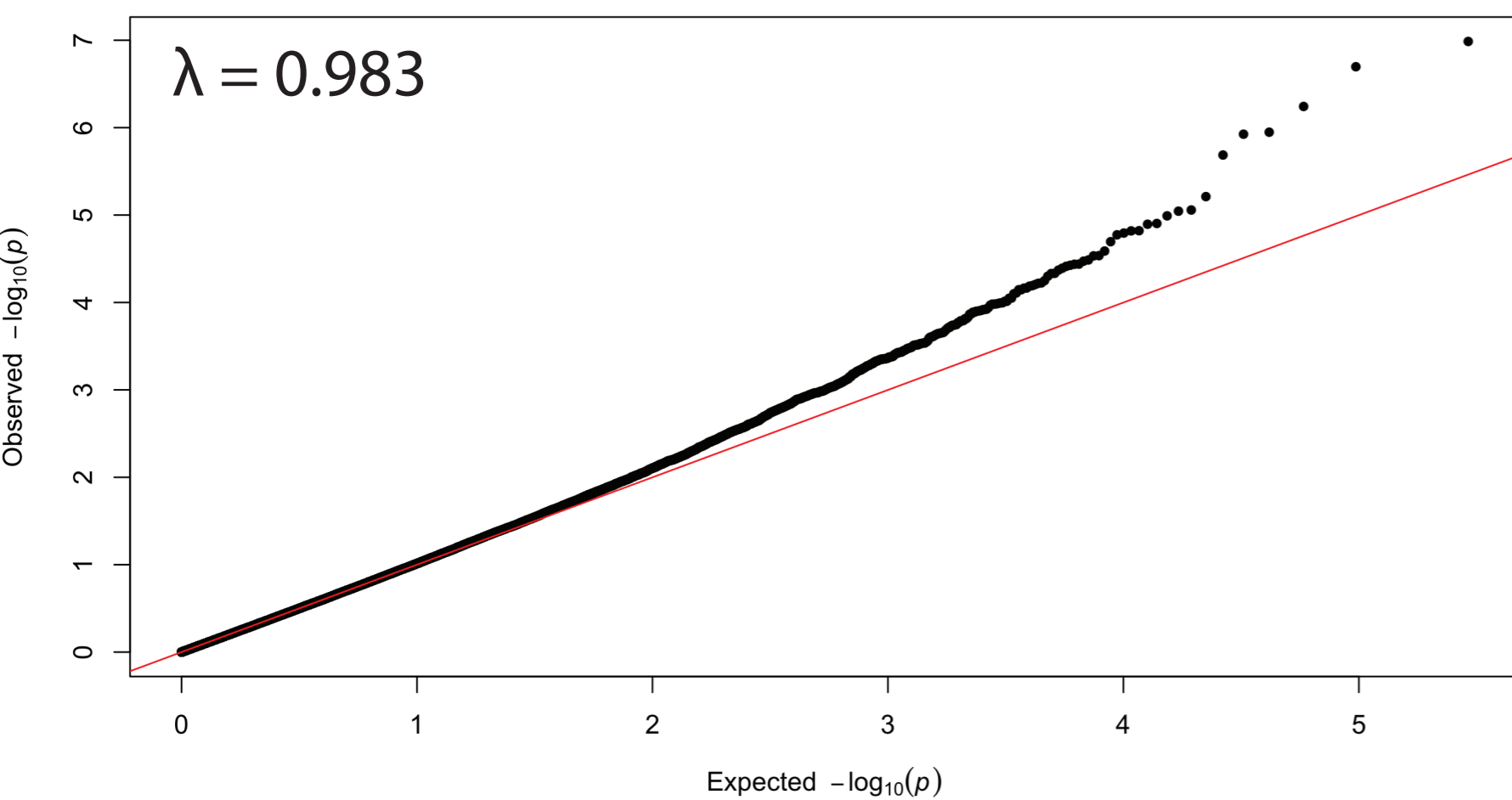

# D. Gestation Length

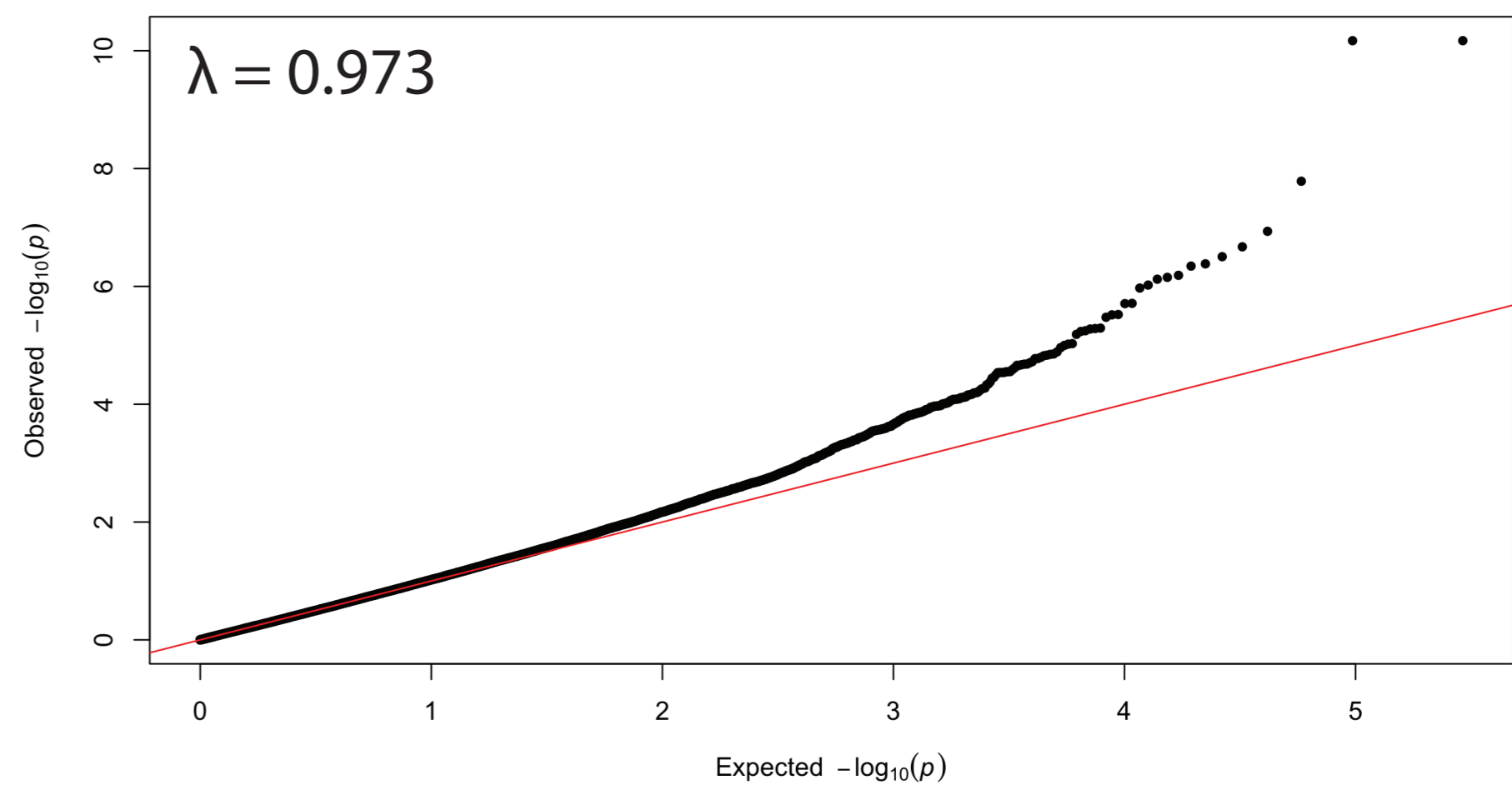

Supplement: eoz015_Supplementary_Data [file eoz015_supplementary_data.zip › Smith_etal_FigS3_QQpanel.pdf]
